# Asian Musk Shrew as a Reservoir of Rat Hepatitis E Virus, China

**DOI:** 10.3201/eid1908.130069

**Published:** 2013-08

**Authors:** Dawei Guan, Wei Li, Juan Su, Ling Fang, Naokazu Takeda, Takaji Wakita, Tian-Cheng Li, Changwen Ke

**Affiliations:** Center for Disease Control and Prevention of Guangdong Province, Guangzhou, China (D. Guan, W. Li, J. Su, L. Fang, C. Ke);; Osaka University, Osaka, Japan (N. Takeda);; National Institute of Infectious Diseases, Tokyo, Japan (T. Wakita, T.-C. Li)

**Keywords:** Suncus murinus, Asian house shrew, Asian musk shrew, family Soricidae, order Soricomorpha, rat, rat hepevirus, rat hepatitis E virus, Hepeviridae, hepevirus, viruses, reservoir, China, epidemiology

**To the Editor:** Rat hepatitis E virus (HEV), a member of genus *Hepevirus* in the family *Hepeviridae*, was first detected in Norway rats in Germany in 2010 (*1**, **2*). Since then, this rat HEVhas been detected in multiple wild rat species in the United States, Vietnam, Germany, and Indonesia (*3**–**7*). Studies have shown that rat HEV failed to infect rhesus monkeys and pigs, suggesting that rat HEV is restricted to its natural host (*6**, **8*). However, it is not known whether animals other than rats are susceptible to rat HEV.

The Asian musk shrew (*Suncus murinus*), also called the Asian house shrew, is a small mole-like mammal belonging to the family *Soricidae* (order Soricomorpha), and wild rats are classified in the family *Muridae* (order Rodentia). Musk shrews originated from the Indian subcontinent and are now found from southern Asia and Afghanistan to the Malay Archipelago and southern Japan. These shrews are commensal rodents, commonly found living in human households. We previously showed that rat HEV infection frequently occurs in wild rats in Zhanjiang City, Guangdong Province, China (*9*). Asian musk shrews share this same environment; thus, they can be exposed to rat HEV derived from wild rats.

To determine whether Asian musk shrews are a reservoir for rat HEV, we examined 260 shrews (112 males, 148 females) that were trapped in Zhanjiang City during December 2011–September 2012. Of the 260 trapped shrews, 147 were from Mazhang District (23 from a pig farm and 124 from the villages of Chiling, Chofa, Beigou, Huangwai, Houyang, and Nanpan) and 113 were from Chikan District.

Blood samples were collected from the shrews, and serum was separated by centrifugation (2,500 × *g* for 20 min at 4°C), and stored at −80°C until use. We tested the serum samples for the presence of HEV IgG and IgM antibodies by using an ELISA based on rat HEV-like particles, as described (*3*). Of the 260 samples, 27 (10.4%) were HEV IgG positive and 12 (4.6%) were HEV IgM positive. Of these, 3 IgG-positive and 1 IgM-positive samples were among the 113 samples (2.7% and 1.0%, respectively) collected from shrews in Chikan District, and 24 IgG-positive and 11 IgM-positive samples were among the 147 samples (16.3% and 7.5%, respectively) collected from shrews in 6 villages (124 total samples) and the pig farm (23 total samples) in Mazhang District. The IgG-positive rate was higher for shrews from Mazhang District than for those from Chikan District (p<0.05); the rates of IgM-positivity did not differ significantly. The IgG-positive rate among the 6 villages varied substantially (8.3%–71.4%) (Technical Appendix Table 1). The IgG-positive rates were 11.6% (13/112) in male and 9.5% (14/148) in female shrews, respectively; the difference in rates between the sexes was not statistically significant.

A total of 12 IgM-positive serum samples were selected for HEV RNA testing by nested broad-spectrum reverse transcription PCR (*2*); results for 5 were positive (Technical Appendix Table 2). The length of the nested reverse transcription PCR products was 334 nt. After the primer sequences were removed, we sequenced the remaining 281 nt corresponding to nt 4107–4387 in the C-terminal open reading frame 1 of the rat HEV genome (GU345042) (GenBank accession nos. KC473527–KC473531). Phylogenetic analysis indicated that the 5 HEV isolates were all classified into the same group as rat HEV and clearly separated into 2 clusters, A and C. Cluster A isolates were further divided into 2 subclusters, sub-A1 (CHZ-sRat-E-1107) and sub-A2 (CHZ-sRat-E-1133) (Figure). Strains CHZ-sRat-E-739, CHZ-sRat-E-1086, and CHZ-sRat-E-1129 belong to cluster C. These findings are of limited precision because of the shot sequence that was analyzed, and, thus, they may not be predictive of results obtained with complete genomes.

**Figure:**
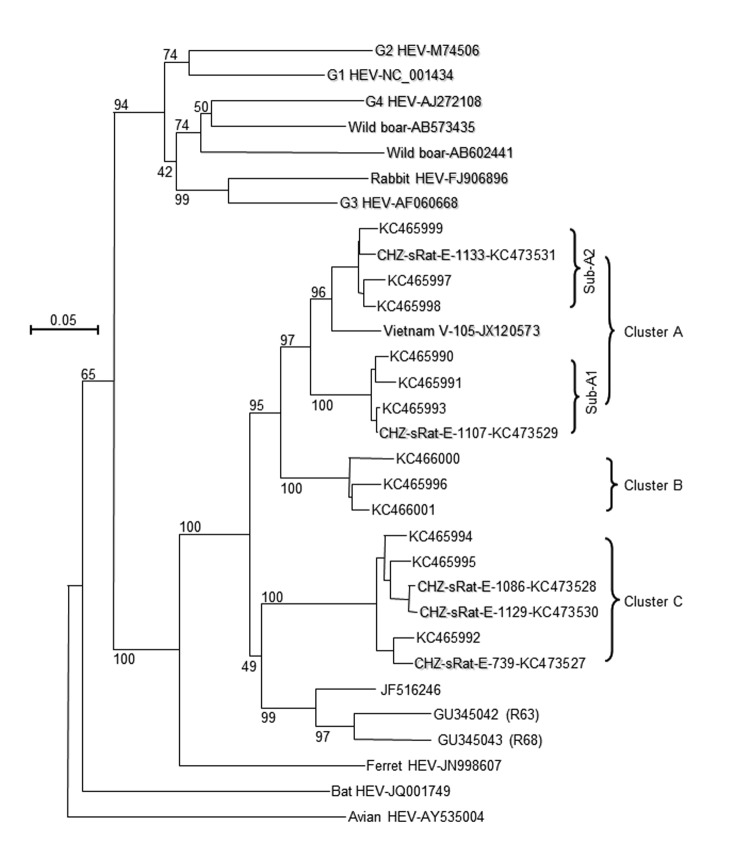
Phylogenetic analysis of rat hepatitis E virus (HEV) isolated from Asian musk shrews (*Suncus murinus*) in Zhanjiang City, China. Nucleic acid sequence alignment was performed by using ClustalX 1.81 (www.clustal.org). The genetic distance was calculated by using the Kimura 2-parameter method. The phylogenetic tree, with 1,000 bootstrap replicates, was generated by the neighbor-joining method based on the partial sequence (281 nt) of HEV open reading frame 1 of genotype 1–4, wild boar, rabbit, ferret, bat, avian, and rat HEV isolates. The scale bar indicates nucleotide substitutions per site.

Rat HEV isolated from the *S. murinus* shrews shared 77.4%–99.6% nt sequence identity with other rat HEV strains; the sequences were especially similar to those of HEV isolates from wild rats in this area (GenBank accession nos. KC465990–KC466001) (Technical Appendix Table 3). In addition, nucleotide sequences from subcluster A1 and A2 and cluster C rat and shrew strains shared 97.5%–99.6%, 96.8%–97.2%, and 94.0%–97.5% identity, respectively (Technical Appendix Table 3). These results indicate that rat HEV infection occurs in *S. murinus* shrews and that these rodents are a reservoir for rat HEV.

Evidence indicates that rat HEV may be capable of inducing an immune response in humans; thus, this virus may be relevant to the epidemiology of HEV in humans (*10*). A key step in understanding this epidemiology is to know the reservoirs of rat HEV, especially reservoirs like *S. murinus* shrews, which live in close proximity to humans.

Technical Appendix Data for 5 rat hepatitis E virus (HEV) isolates from Asian musk shrews (*Suncus murinus*) and IgG and IgM positivity rates among shrews trapped during December 2011–September 2012 in Guangdong Province, China, and comparison of nucleotide sequence identities for rat HEV strains from wild rats and Asian musk shrews.

## References

[1] Johne R, Heckel G, Plenge-Bönig A, Kindler E, Maresch C, Reetz J, Novel hepatitis E virus genotype in Norway rats, Germany. Emerg Infect Dis. 2010;16:1452–5. 10.3201/eid1609.100444 20735931 PMC3294985

[2] Johne R, Plenge-Bonig A, Hess M, Ulrich RG, Reetz J, Schielke A. Detection of a novel hepatitis E–like virus in faeces of wild rats using a nested broad-spectrum RT-PCR. J Gen Virol. 2010;91:750–8. 10.1099/vir.0.016584-0 19889929

[3] Li TC, Yoshimatsu K, Yasuda SP, Arikawa J, Koma T, Kataoka M, Characterization of self-assembled virus-like particles of rat hepatitis E virus generated by recombinant baculoviruses. J Gen Virol. 2011;92:2830–7. 10.1099/vir.0.034835-0 21865442 PMC3352569

[4] Li TC, Ami Y, Suzaki Y, Yasuda SP, Yoshimatsu K, Arikawa J, Characterization of full genome of rat hepatitis E virus strain from Vietnam. Emerg Infect Dis. 2013;19:115–8 . 10.3201/eid1901.121007 23260149 PMC3558001

[5] Mulyanto, Depamede SN, Sriasih M, Takahashi M, Nagashima S, Jirintai S, et al. Frequent detection and characterization of hepatitis E virus variants in wild rats (*Rattus rattus*) in Indonesia. Arch Virol 2013;158:87–96. **PMID 22983110** 10.1007/s00705-012-1462-0 22983110

[6] Purcell RH, Engle RE, Rood MP, Kabrane-Lazizi Y, Nguyen HT, Govindarajan S, Hepatitis E virus in rats, Los Angeles, California, USA. Emerg Infect Dis. 2011;17:2216–22. 10.3201/eid1712.110482 22172320 PMC3311208

[7] Johne R, Dremsek P, Kindler E, Schielke A, Plenge-Bonig A, Gregersen H, Rat hepatitis E virus: geographical clustering within Germany and serological detection in wild Norway rats (*Rattus norvegicus*). Infect Genet Evol. 2012;12:947–56. 10.1016/j.meegid.2012.02.021 22554648

[8] Cossaboom CM, Cordoba L, Sanford BJ, Pineyro P, Kenney SP, Dryman BA, Cross-species infection of pigs with a novel rabbit, but not rat, strain of hepatitis E virus isolated in the United States. J Gen Virol. 2012;93:1687–95. 10.1099/vir.0.041509-0 22535776 PMC3541760

[9] Li W, Guan D, Su J, Fang L, Takeda N, Wakita T, High prevalence of rat hepatitis E virus in wild rats in China. Vet Microbiol. 2013;13:187–9 . 23623690 10.1016/j.vetmic.2013.03.017

[10] Dremsek P, Wenzel JJ, Johne R, Ziller M, Hofmann J, Groschup MH, Seroprevalence study in forestry workers from eastern Germany using novel genotype 3 and rat hepatitis E virus–specific immunoglobulin G ELISAs. Med Microbiol Immunol (Berl). 2012;201:189–200 . 10.1007/s00430-011-0221-2 22179131

