# Supplementary material for: Asian Musk Shrew as a Reservoir of Rat Hepatitis E Virus, China
Source: Emerg Infect Dis. 2013 Aug;19(8):1341–3. doi: 10.3201/eid1908.130069 (PMC3739524; doi:10.3201/eid1908.130069)
Supplement: Technical Appendix — Data for 5 rat hepatitis E virus (HEV) isolates from Asian musk shrews (Suncus murinus) and IgG and IgM positivity rates among shrews trapped during December 2011–September 2012 in Guangdong Province, China, and comparison of nucleotide sequence identities for rat HEV strains from wild rats and Asian musk shrews. [file 13-0069-Techapp-s1.pdf]

Article DOI: http://dx.doi.org/10.3201/eid1908.130069

Asian Musk Shrew as a Reservoir of Rat Hepatitis E Virus, China

Technical Appendix

Technical Appendix Table 1. Rat hepatitis E virus IgG and IgM positivity rates among Asian musk shrews (*Suncus murinus*) trapped in Zhanjiang City, Guangdong Province, China, December 2011–September 2012

	% (no. positive/no. tested)	
Geographic region	IgG	IgM
Total	10.4 (27/260)	4.6 (12/260)
Chikan district	2.7 (3/113)	1.0 (1/113
Mazhang district	16.3 (24/147)	7.5 (11/147)
Huangwai village	13.3 (2/15)	0 (0/15)
Houyang village	71.4 (5/7)	28.6 (2/7)
Nanpan village	23.8 (5/21)	9.5 (2/21)
Chiling village	8.3 (1/12)	0 (0/12)
Beigou village	16.7 (1/6)	16.7 (1/6)
Chaofa village	9.5 (6/63)	7.9 (5/63)
Pig farm	17.4 (4/23)	4.3 (1/23)

Table 2. Rat hepatitis E virus–positive strains isolated from Asian musk shrews (*Suncus murinus*) trapped in Zhanjiang City, Guangdong Province, China, December 2011–September 2012*

			Village where	Optical density value		Cluster or	GenBank
Isolate name	Date of sampling	Sex	trapped	IgG	IgM	subcluster	accession no.
CHZ-sRat-E-739	2012 May14	M	Nanpan	2.254	0.492	C	KC473527
CHZ-sRat-E-1086	2012 Sep 19	F	Chaofa	2.767	0.289	C	KC473528
CHZ-sRat-E-1107	2012 Sep 19	F	Chaofa	0.123	0.253	A1	KC473529
CHZ-sRat-E-1129	2012 Sep.20	F	Chaofa	0.104	0.561	C	KC473530
CHZ-sRat-E-1133	2012.Sep 20	M	Chaofa	0.101	0.505	A2	KC473531

*Positive results were obtained by reverse transcription PCR.

Table 3. Percent shared nucleotide sequence identities between rat hepatitis E virus strains from rats and Asian musk shrews (*Suncus murinus*) trapped in Zhanjiang City, Guangdong Province, China, December 2011–September 2012

Rat HEV strain by GenBank accession no.	% Identity with rat hepatitis E virus strains from Asian musk shrews				
	KC473529	KC473531	KC473528	KC473530	KC473527
KC465999	91.4	97.2	79.3	78.9	80.0
KC465997	89.3	96.8	79.3	78.9	80.0
KC465998	89.6	97.2	80.0	79.6	80.7
KC465990	97.5	89.3	79.1	78.7	79.4
KC465991	97.5	87.9	78.2	77.9	78.6
KC465993	99.6	90.0	78.6	78.2	78.9
KC466000	83.3	84.6	77.5	78.3	77.6
KC465996	85.1	85.0	79.3	80.0	78.9
KC466001	86.1	87.5	79.3	80.0	78.9
KC465994	79.6	81.1	97.5	96.4	95.4
KC465995	80.0	81.4	97.2	96.1	96.4
KC465992	79.3	81.1	94.0	94.3	97.2
JX120573	90.3	93.2	81.0	80.6	81.0
GU345042	77.5	80.7	80.3	79.9	79.2
GU345043	74.6	77.9	78.9	78.9	78.9
KC473529	100.0	90.4	78.9	78.6	79.3
KC473531	90.4	100.0	80.0	79.6	80.7
KC473528	78.9	80.0	100.0	98.9	95.7
KC473530	78.6	79.6	98.9	100.0	96.1
KC473527	79.3	80.7	95.7	96.1	100.0
